# Editorial: Innate Immunity in Aquatic Vertebrates

**DOI:** 10.3389/fimmu.2019.02959

**Published:** 2019-12-19

**Authors:** Stephanie DeWitte-Orr, Eva-Stina Edholm, Leon Grayfer

**Affiliations:** ^1^Department of Biology, Wilfrid Laurier University, Waterloo, ON, Canada; ^2^Faculty of Biosciences, Fisheries and Economics, Norwegian College of Fishery Science, University of Tromsø-The Arctic University of Norway, Tromsø, Norway; ^3^Department of Biological Sciences, George Washington University, Washington, DC, United States

**Keywords:** innate immunity, fish, amphibia, antiviral, Research Topic

The alarming growth of the world's population is putting ever-greater demand on agricultural industries and is manifesting in environmentally detrimental consequences. While the development of better aquaculture practices presents a promising means of meeting the needs of this population growth, overcrowding in aquaculture, climate change, and habitat destruction are resulting in the emergence of new and opportunistic infections within farmed and wild aquatic vertebrate species, often to the detriment of these animals. The prevention and circumvention of these infections and die-offs requires much greater understanding of the mechanisms by which these animals' immune systems develop, recognize, and respond to distinct pathogens. Indeed, we already know that while animals like cartilaginous/bony fish and amphibians exhibit hallmark components associated with mammalian immunity, they also provide examples of novel strategies for immune cell development and antimicrobial defenses. As these organisms possess arguably less developed adaptive immune responses, they rely more heavily on their innate immunity to control infiltrating pathogens. In turn, these animals reside in vastly distinct environments to those within which (the much more extensively characterized) mammalian immune system has evolved, so it is not surprising that aquatic vertebrates possess many intriguing immunological differences from terrestrial animals.

It is by gaining greater insight into these immune processes that we may hope to better our aquacultural practices and combat the devastating effects of human activities on aquatic animal communities around the globe. Toward this end and through this collection of 17 articles, which include both original research as well as comprehensive reviews, we coalesce recent advances in the current understanding of the innate immune responses of aquatic vertebrates.

## Antifungal Defenses

Aquatic vertebrates reside within pathogen-rich environments, with their skin mucosa representing an important barrier to these pathogens, but also a means of pathogen entry. The global amphibian declines resulting from the *Batrachochytrium dendrobatidis* and *Batrachochytrium salamandrivorans* chytrid fungi infections of amphibian skins is an important example of this skin mucosa-pathogen interface. The comprehensive review by Varga et al. underlines the importance of the amphibian skin as an innate immune barrier to aquatic pathogens, discusses the anatomy and cell (immune and non-immune) composition to of the amphibian skin, and focuses on the skin pattern recognition receptors (PRR) and antimicrobial peptide responses therein.

In turn, Grogan et al. provide an extensive overview of the documented and anticipated amphibian immune responses against chytrid pathogens, covering topics such as the determinants of skin anti-fungal protection, constitutive skin immune defenses, innate immune recognition, and the ensuing innate immune and adaptive immune responses to fungal pathogens. Grogan et al. evaluate and discuss the presumed and potential roles of pathogen detection, immune suppression, fungal immune evasion, immunological successes, and possible failures as well as immunopathology in the context of chytridiomycosis.

## Pathogen Recognition Responses

Aquatic animals are subject to very different pathogen pressures to those that have shaped the terrestrial immune response, and yet many aspects of their innate immune armamentarium are conserved. While these animals possess many of the same PRR genes as terrestrial mammals, they also encode species-specific pathogen receptors and may well-utilize the mammalian PRR homologs in distinct ways.

As an example of the above and unlike mammals, aquatic animals are notoriously insensitive to the lipopolysaccharide (LPS) and presumably have evolved distinct/complementary means for LPS detection. In this respect, Bi et al. demonstrate that the nucleotide-binding oligomerization domain-containing protein 1 (NOD-1), which is best known as a receptor for intact bacteria-derived peptidoglycan; in fish may also serve as a means for recognizing intracellular LPS, resulting in the canonical activation of NF-κB signaling pathway and the ensuing proinflammatory response.

Across vertebrates, β-glucan carbohydrates present on the surfaces of an array of pathogens also represent important PRR ligands and therefore a means of pathogen recognition. While the mammalian Dectin-1 receptor (member of C-type lectin receptor family; CLR) is the best characterized β-glucan PRR, this gene has to date not been clearly annotated in fish genomes, although fish such as carp have been shown to recognize this pathogen associated molecular pattern (PAMP). Petit et al. demonstrate that in response to β-glucan stimuli, common carp macrophages undergo cell signaling pathway that are characteristic of CLR activation. Moreover, using a number of bioinformatics approaches, this study identifies several putative carp CLR- β-glucan receptors, some of which possess gene synteny and structural similarities to the mammalian Dectin-1. This presumably highlights both the convergence and the diverged evolution of the fish and terrestrial mammal innate immune pathogen recognition.

## Granulocyte Development and Recruitment

In terrestrial mammals, granulocytes are amongst the first cells to respond to infiltrating pathogens as well as the most represented immune populations in circulating blood. While the kinetics of the aquatic vertebrate immune infiltration of infected tissues appear to correspond to those of mammals, the mechanisms by which fish and frogs generate and recruit their granulocyte populations differ from what is seen in mammals.

Where the granulocyte colony-stimulating factor (G-CSF) is the principal driver of granulopoiesis, it is interesting to consider that while mammals possess a single G-CSF, teleosts encode multiple G-CSF isoforms. Intriguingly, Katakura et al. demonstrate the presence in the common carp genome of four G-CSF paralogs (*g-csfa1* and *g-csfa2*; *g-csfb1* and *g-csfb2*), which exhibit distinct expression across fish tissues, leukocytes, and following immune stimulation. Moreover, while the common carp G-CSFa1 and G-CSFb1 both elicit neutrophil chemotaxis and proliferation of kidney cells, only G-CSFb1 promotes neutrophil-lineage differentiation of head kidney cells.

In turn, while mammals possess a single CXCL8 chemokine bearing the ELR motif, characteristic of pro-inflammatory granulocyte chemokines, Koubourli et al. demonstrate that the amphibian *Xenopus laevis* encode two CXCL8s, one of which possesses the ELR motif and appears to be involved in inflammatory responses, and the other lacking this motif and being involved in the recruitment of healing/immunosuppressive granulocytes.

## Antiviral Immunity

Aquatic animals are important models for the study the converged and divergent evolution of vertebrate innate and antiviral immunity. As the interferon (IFN) cytokine responses represents the cornerstone of vertebrate antiviral defenses, it is exciting to consider that while the emergence of type III IFN responses was thought to emerge with tetrapods, Redmond et al. show that cartilaginous fish encode both type I and type III IFNs, thus instead suggesting the loss of this cytokine family in bony fish and its reemergence in amphibians.

Aquatic habitats teem with viral pathogens so it is perhaps not surprising that aquatic vertebrates have evolved elaborate antiviral defenses, several of which are discussed here. Amongst these, Lazarte et al. comprehensively review the current understanding of the fish Mda5 antiviral PRR and its roles in fish recognition of intracellular viral and bacterial pathogens, the initiation of the fish type I IFN response and the consequences of the activation of this receptor to bony fish immunity. Chen et al. report on the characterization of a fish TANK-binding kinase 1, which appears to be an important regulator of fish IFN response. Xu et al. report on a fish-specific PKR analog, protein kinase Z, which activates a number of hallmark antiviral signaling components and elicits the expression of IFN. Eslamloo et al. characterize the cod Viperin antiviral effector gene, model its protein architecture in comparison to mammalian Viperins and examine cod Viperin expression during cod development, following immune stimulation of cod macrophages and in conjunction with a panel of immune inhibitors, thereby elucidating possible regulatory pathways for this gene. Zhang et al. report on the characterization of the grouper cholesterol 25-hydroxylase (CH25H) IFN-induced gene including *in silico*, expression, subcellular localization, and functional analyses of the grouper CH25H in the context of Singapore grouper iridovirus and red-spotted grouper nervous necrosis virus infections. Lastly, Li et al. describe the antiviral roles of the orange-spotted grouper autophagy-related gene-5 (Atg5) in the context of red-spotted grouper nervous necrosis virus and Singapore grouper iridovirus infections.

## Innate-Like B Cells

Teleost fish appear to possess greater numbers of innate-like phagocytic B cells than mammals and thus, understanding the roles of these cells during immune responses and how they are affected by vaccination is key to better fish vaccine development. To this end Wu et al. show that while the Nile tilapia IgM^lo^ B cells (resembling plasma-like cells) possess decreased phagocytic activity compared to the naïve/mature-like IgM^hi^ B cells, suggesting that B cell differentiation may cause the decrease in phagocytic capacities of bony fish B cells. In turn, this work may indicate that the evolution of more specialized (further differentiated) B cell responses in mammals compared to bony fish, may have come at the expense of decreased phagocytic capacities of these cells, in favor of antibody-production. Concurrently, Kordon et al. challenge catfish kidney-derived B cells with wild type and vaccine strains of *Edwardsiella ictaluri*, showing that both bacterial strains are phagocytosed by the B cells, eliciting antimicrobial activity but also inducing apoptosis in these fish B cells.

## Fish Health and Vaccination

While nanoparticles are being increasingly utilized in many industries, the consequences of their bioaccumulation within aquatic environments remains poorly addressed. Accordingly, Torrealba et al. review the use of fish innate immune markers as an auspicious means for assessing fish health following nanoparticle exposure.

Greater insights into aquatic animal immune responses lead to the development of better vaccination strategies for these animals and several of the manuscripts in this Research Topic exemplify this notion. For example, Braden et al. have utilized the previously documented salmon immune responses to *Aeromonas salmonicida* spp. *salmonicida* (*Asal*) and several vaccines to demonstrate in the Arctic charr (an emerging aquacultural species) that efficacies of vaccine-based protection against *Asal* depend on the upregulation and control of fish baseline humoral responses, including factors such as complement and coagulation factors, acute phase-proteins, and iron hemostasis proteins.

## Concluding remarks

The primary articles and reviews featured in this Research Topic are great examples of the exciting new research being conducted on innate immunity of aquatic vertebrates. With every new article, we gain greater understanding of the interesting and often unique mechanisms governing these animals' antimicrobial defenses. In turn, these studies will pave the way toward the development of better aquacultural practices, aquatic habitat preservation and remediation as well as a deeper understanding of the evolution of vertebrate immune responses.

## Author Contributions

All authors listed have made a substantial, direct and intellectual contribution to the work, and approved it for publication.

### Conflict of Interest

The authors declare that the research was conducted in the absence of any commercial or financial relationships that could be construed as a potential conflict of interest.

